# Aquaculture related humpback whale entanglements in coastal waters of British Columbia from 2008–2021

**DOI:** 10.1371/journal.pone.0297768

**Published:** 2024-03-20

**Authors:** Rhea L. Storlund, Paul E. Cottrell, Brendan Cottrell, Myron Roth, Taylor Lehnhart, Heindrich Snyman, Andrew W. Trites, Stephen A. Raverty

**Affiliations:** 1 Marine Mammal Research Unit, Institute for the Oceans and Fisheries, University of British Columbia, Vancouver, BC, Canada; 2 Department of Zoology, University of British Columbia, Vancouver, BC, Canada; 3 Fisheries and Oceans Canada, Fisheries and Aquaculture Management, Vancouver, BC, Canada; 4 Applied Remote Sensing Lab, Department of Geography, McGill University, Montreal, QC, Canada; 5 Aquaculture and Marine Fisheries, BC Ministry of Agriculture and Food, Victoria, BC, Canada; 6 Animal Health Laboratory, Laboratory Services Division, University of Guelph, Kemptville, ON, Canada; 7 Animal Health Center, Ministry of Agriculture, Abbotsford, BC, Canada; Annamalai University, INDIA

## Abstract

Over the past two decades, increasing numbers of humpback whales have been returning to feed in the inshore waters of British Columbia (BC) where marine aquaculture farms are situated. This has led to growing concerns that the presence of aquaculture farms may pose an entanglement threat to humpback whales. However, it is not known whether aquaculture facilities attract humpback whales, or whether there are factors that increase the likelihood of humpback whale, becoming entangled and dying. We examined eight reports of humpback whales interacting with Atlantic salmon farms in BC from 2008 to 2021 to evaluate the conditions that may have contributed to their entanglements. Of the eight entangled humpbacks, three individuals died and five were successfully disentangled and released. All were young animals (1 calf, 7 subadults). Multiple factors were associated with two or more of the reported incidents. These included facility design, environmental features, seasonality, humpback whale age, and feeding behaviour. We found that humpback whales were most commonly entrapped in the predator nets of the aquaculture facilities (6/8 incidents), and were less often entangled in anchor support lines (2/8). The presence of salmon smolts did not appear to be an attractant for humpback whales given that half of the reported entanglements (4/8) occurred at fallowed salmon farms. Almost all of the entanglements (7/8) occurred in late winter (prior to the seasonal return of humpbacks) and during late fall (after most humpbacks have migrated south). Overall, the number of humpback whales impacted by fish farms was small compared to the numbers that return to BC (> 7,000) and accounted for <6% of all types of reported entanglements in BC. Human intervention was required to release humpback whales at fish farms, which points to the need to have well-established protocols to minimize entanglements and maximize successful releases.

## Introduction

Humpback whales are present in British Columbia (BC) year-round, but their peak abundance occurs from April–November as they migrate from calving grounds in Japan, Hawaii, and Mexico to feeding grounds throughout the northeastern Pacific [1, 2]. In BC, humpback whales have significant ecological, economic, and socio-cultural impacts. Ecologically, they directly benefit the local ecosystem by contributing carbon to the marine life cycle, adding nutrients through excretion, mixing nutrients by diving and swimming through areas that may not have strong currents, and providing an abundance of nutrients to bottom-dwelling animals when they die and sink to the ocean floor [3]. In addition, they are recognized as an indicator of ecosystem health because their diets reflect oceanographic and ecological conditions, and environmental variability such as changes in water temperature [4, 5]. Economically, their presence is a major contributor to the whale watching industry which generated 27 million dollars in 2008 in direct revenue and 91 million dollars indirectly [6]. Humpback whales are also culturally significant to coastal First Nations who historically hunted them for subsistence [1].

The BC coast is an area of high productivity that attracts and sustains many populations of marine mammals. Humpback whales are often seen in high vessel-traffic areas such as Dixon Entrance, Hecate Strait, and the entrance to Juan de Fuca Strait in the spring and summer months [7, 8]. They are primarily observed on the continental shelf [9], and show preference for shelf waters between 50 and 200 m depth, especially the 100 m contour [7, 10]. In BC, many economically important human activities, including ecotourism, commercial and sport fishing, and aquaculture [11, 12] occur in the same locations that humpback whales frequent. Unfortunately, this overlap in space use increases the risk of injury and even death to humpback whales by increasing their exposure to anthropogenic activity.

The number of humpback whales in BC is increasing [13]. Population estimates increased from 92 to 526 humpback whales from 1994 to 2014 in southern BC and Washington [13]. An estimated 279 humpback whales were present in the Salish sea in 2018, and a total of 7,030 humpback whales were present throughout BC waters (Salish sea, North coast, and offshore combined) [8]. Increased whale presence creates more opportunities for interaction with industry, vessels, and aquaculture because fishery operations occupy the same geographical areas as humpback whales and often target the same marine life for consumption [14]. In 2022, along the BC coast, there were 484 shellfish marine tenures and 86 finfish tenures [15] with approximately 6,776 hectares of shellfish farms and 4,988 hectares of finfish farms [16], comprised predominantly of Atlantic salmon [15]. Salmon farms in BC are anchored in near-shore waters and consist of a cage array with net pens where the salmon are housed, and a surrounding predator net to reduce consumption of stocked salmon by predators such as seals and sea lions [11]. Prior to 2017, these facilities may have also included ropes used to reposition anchors called anchor support lines. Though they share many similarities, each facility has a unique setup that must be engineered to deal with environmental features such as tides, currents, prevailing winds, and bathymetry. Historically, little attention may have been afforded to potential interactions between fish farms and marine mammal species [17, 18].

Humpback whales may be at increased risk of entanglement in aquaculture gear as they frequent inshore waters and coastal inlets where aquaculture farms are preferentially situated. Entanglements or entrapments and breach of fish farm perimeters may result in injury and death of humpback whales, damage to fish farm gear (e.g., large holes in the predator or containment nets), fish release, predation, and negative publicity associated with harming a humpback whale and escapement of non-native salmonid species. Institution of appropriate design, management, and response protocols should mitigate these types of interactions and benefit aquaculture production and humpback whales. Determining how to accomplish these measures requires an in-depth assessment of previous interactions. What attracts humpback whales to aquaculture facilities and contributes to humpback whale entanglements is currently unknown.

Humpback whales are important contributors to BC’s marine ecosystem. In addition to vessel and propeller strikes [9], and entanglements with fishing gear [19], humpback whales appear to be at risk of injury and death from interactions with aquaculture facilities. In this paper, we describe eight events with three fatalities where humpback whales were entangled or entrapped in the net pens, predator nets, or anchor support lines of BC aquaculture facilities, speculate on the factors that contribute to these interactions, and suggest ways for aquaculture facilities to mitigate adverse large whale interactions.

## Materials and methods

The case definition for inclusion in this study was a report to the BC Marine Mammal Response Network (BCMMRN) that clearly described a situation where a humpback whale was found within a net pen, predator net, or entangled in an anchor support line at a marine finfish aquaculture facility. Information regarding humpback whale interactions with aquaculture facilities in BC was collected from the BCMMRN, BC Ministry of Agriculture Animal Health Centre, and aquaculture companies involved in the interactions.

In those incidents with a history of presumptive, suspected, or confirmed net entanglement or recovery of a carcass from an aquaculture facility with evidence of net or rope entanglement, a necropsy was performed as part of an enforcement investigation. In the two cases where carcasses were identified, the animals were towed to a location away from the aquaculture site, secured ashore at high tide, and a necropsy was performed by conventional techniques [20]. Morphometric data was collected, lesions associated with the entanglement were photographed, the animals were necropsied, and representative tissues collected for laboratory studies and microscopic evaluation. Results were reviewed by two veterinary pathologists with experience in marine mammal pathology and a final report for each animal was generated.

After developing a case definition and identifying the humpback whale-aquaculture interactions through a review of the BC Marine Mammal Incident Database, farm operators were approached for additional details with regards to enclosure design, environmental and oceanographic features at the farm site, and management schemes. Descriptions or diagrams of the facilities including substrate depth and topography and proximity of the facility to land were collected and compared. Additional details included the use of night-lights and deterrents, such as bubble curtains at the site, and stock size (stage of production) at the time of the incident. Additional data on the production status (active or fallow) was provided by the Department of Fisheries and Oceans Canada (DFO).

Results from the BCMMRN, Animal Health Centre, and aquaculture companies were tabulated, and details of each incident were reviewed. Tables include incident date, farm location, coordinates, interaction type, signalment (humpback whale sex, age, and length), nutritional condition and post-mortem state. Ages and lengths of disentangled whales were estimated by drone or on-board observations. Age classifications were based on body length and proximity to an adult female humpback whale. Calves and subadults were less than 11.0 m total body length, but only calves were observed in proximity to an adult female humpback whale. These age estimates agree with a previous study that classified humpback whales that were 8.0–11.6 m as juveniles [21].

**Fig 1:**
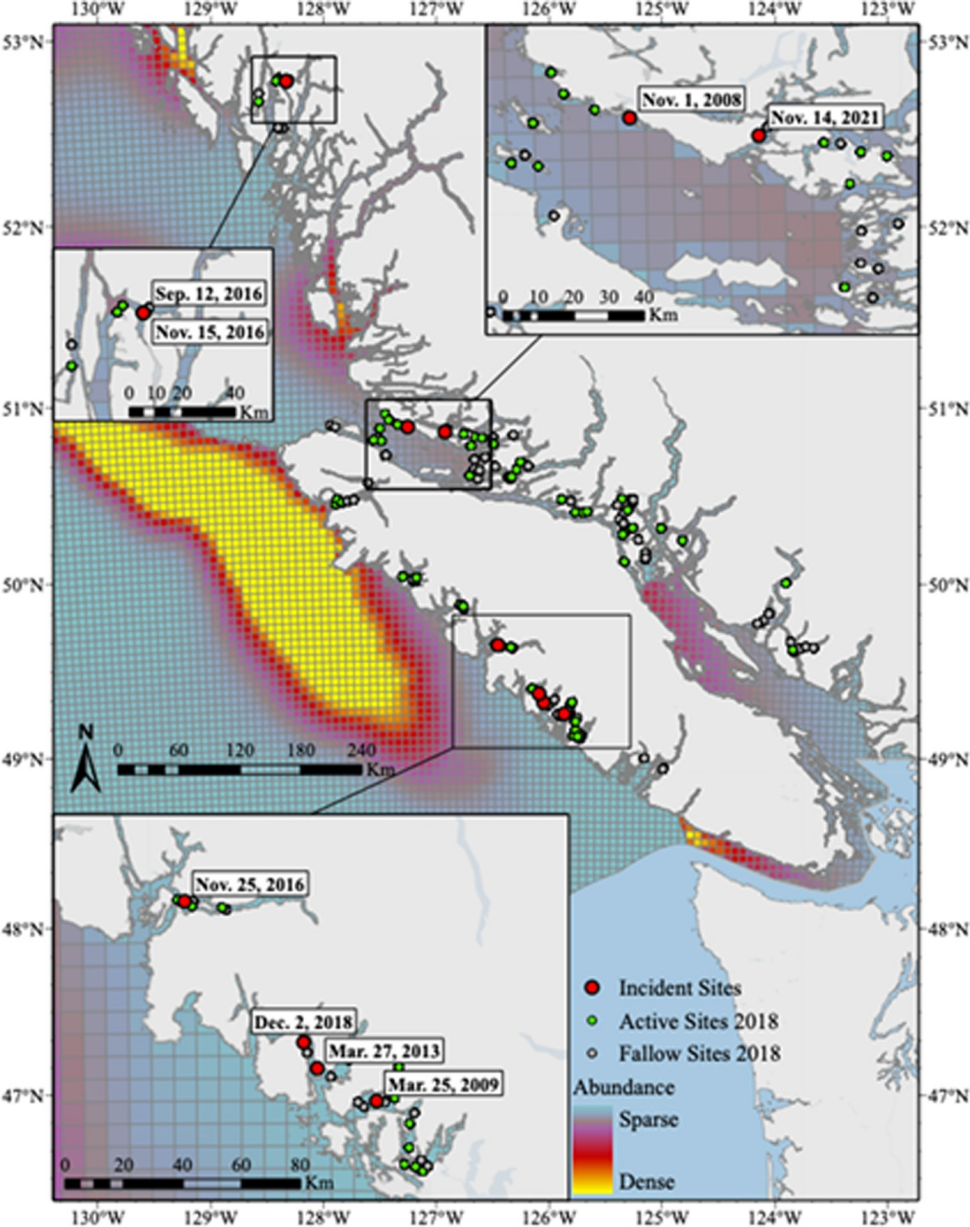
Humpback whale-aquaculture interactions and predicted abundance. Locations of humpback whale-aquaculture interactions (red points) from 2008–2021, active (teal points) and fallow (grey points) aquaculture sites in 2018, and predicted humpback whale abundance based surveys conducted in the summer of 2018 along the British Columbia coast. Reported entanglement locations (in decimal degrees) were mapped using ArcGIS Pro 3.0.2 with public domain Natural Earth base maps (naturalearthdata.com). Spatial density models predicting humpback whale abundance were reproduced from the PRISMM study [8] with permission. The PRISMM study modelled humpback whale abundance separately for the North Coast, Salish Sea, and West Coast of Vancouver Island. On this map, all three models are plotted together despite being based on different inputs. Note that the surveys were not conducted near-shore or up inlets, and therefore likely underestimate humpback whale presence in these areas. See [8] for complete survey and model details.

**Fig 2:**
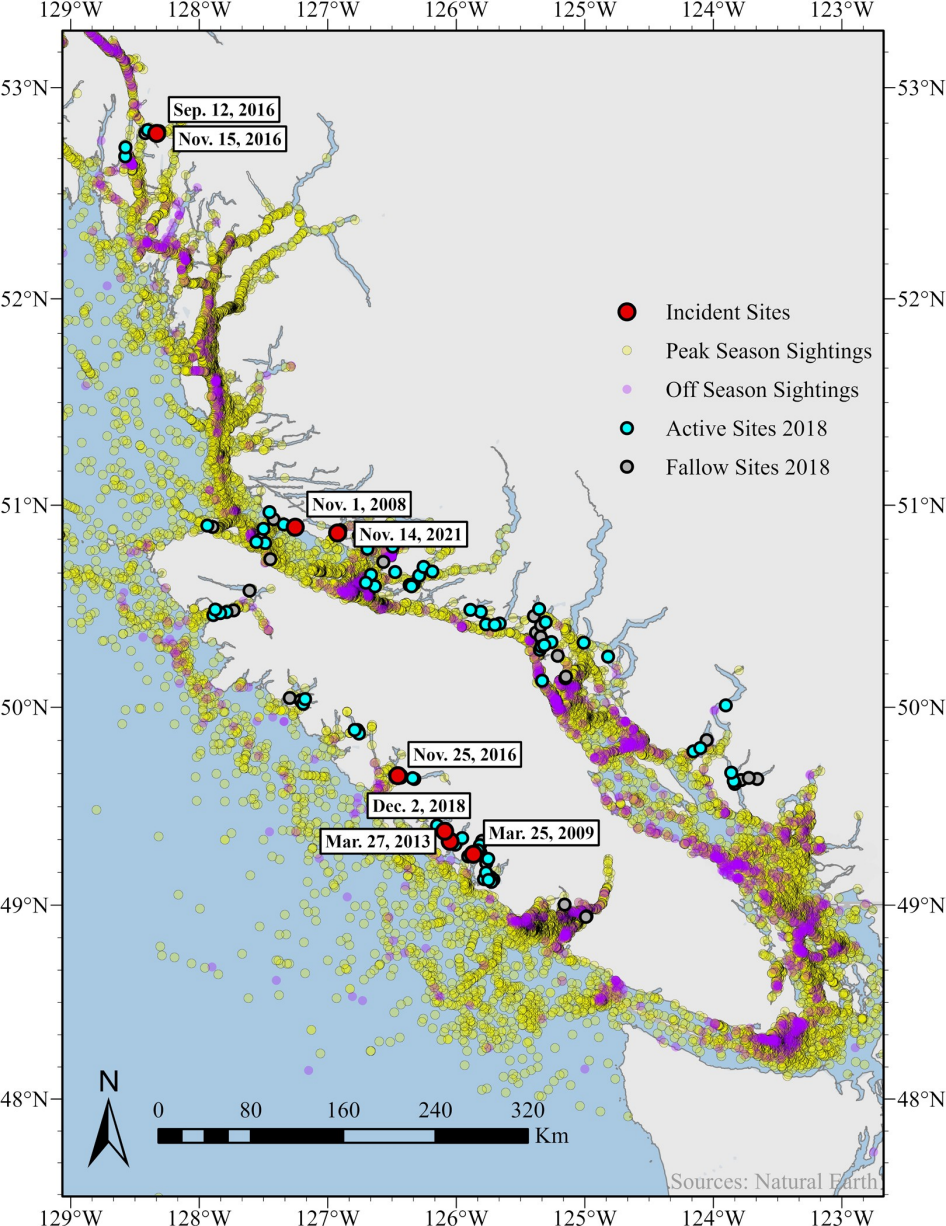
Humpback whale sightings and aquaculture locations. Seasonal sightings of humpback whales from the BC Cetacean Sightings Network are plotted in relation to humpback whale-aquaculture incident locations (red points) and licensed aquaculture sites in 2018. Peak season (yellow points) was defined as April to October and off season (purple points) was defined as November to March. Reported entanglement locations (in decimal degrees) were mapped using ArcGIS Pro 3.0.2 with public domain Natural Earth base maps (naturalearthdata.com). Sightings reports from 1983–2022 are included. Sightings data supplied by the BC Cetacean Sightings Network. Sightings are opportunistic and not corrected for effort.

To assess the spatial overlap between aquaculture facilities and humpback whale habitat in BC, we generated two maps from four data sets (Figs 1 and 2). The first map displays the locations of active and fallow licensed aquaculture facilities in 2018, humpback whale-aquaculture incidents from 2008–2021, and three spatial density models of humpback whale abundance created from data collected in the summer of 2018 (Fig 1) [8]. Spatial densities were modelled based on region and had different model inputs [8], but were mapped altogether because the output, predicted number of whales, was the same. The second map displays the locations of licensed aquaculture facilities in 2018, humpback whale-aquaculture incidents from 2008–2021, and humpback whale sightings supplied by the BC Cetacean Sightings Network (BCCSN) (Fig 2). Humpback whale sightings were grouped into two categories to identify seasonal patterns. Peak season was from April to October, and off season was from November to March based on the timing stated in Ford [2]. Data obtained from the BC Cetacean Sightings Network were collected opportunistically with limited knowledge of the temporal or spatial distribution of observer effort. As a result, absence of sightings at any location does not demonstrate absence of cetaceans. For both maps, aquaculture facility coordinates were supplied by DFO. Maps were generated using ArcGIS Pro 3.0.2 with public domain Natural Earth base maps (naturalearthdata.com).

## Results

Review of incident and stranding data identified eight humpback whale interactions with aquaculture between 2008 and 2021. Each incident is summarized in Table 1. All interactions involved Atlantic salmon farming in sites that were either fallow (*n* = 4) or in active production (*n* = 4). Each interaction involved a single humpback whale and the aquaculture facility. No group or mass entanglements were reported, but in one case, the mother of an entangled calf was observed in the area at the time of the interaction. Five whales were initially observed between the predator and containment nets, two were entangled in anchor support lines, and one was found inside a net pen. Five whales were successfully disentangled and released, two were observed alive (but subsequently died), and one was found dead.

**Table 1:** Descriptions of humpback whale interactions with aquaculture in BC from 2008–2021.

Date	Location	Latitude	Longitude	Water depth (m)	Interaction type	Sex	Age	Length (cm)	Outcome
Nov. 1, 2008	Raynor Site	50.89253	-127.25359	—	Inside net pen	—	Subadult	914	Live release
Mar. 25, 2009	Mussel Rock	49.25925	-125.86762	10–30	Inside predator net before containment nets had been installed	—	Calf	—	Live release
Mar. 27, 2013	Ross Pass	49.32437	-126.04849	30–40	Between containment net and predator net	F	Subadult	910	Dead
Sep. 12, 2016	Lime Point	52.78538	-128.33133	—	Multiple wraps of anchor support line through the oral cavity and baleen	—	—	—	Live release
Nov. 15, 2016	Lime Point	52.78538	-128.33133	—	Single wrap of anchor support line around tail at inactive facility	—	Subadult	935	Dead
Nov. 25, 2016	Gold River	49.65603	-126.45404	70–140	Between containment net and predator net	F	Subadult	1051	Dead
Dec. 2, 2018	Millar Channel	49.37622	-126.09003	40–100	Between containment net and predator net	—	—	—	Live release
Nov. 14, 2021	Wehlis Bay	50.86410	-126.92374	45–80	Inside predator net after containment nets had been removed	—	Subadult	—	Live release

The interactions occurred at 7 different facilities (Table 1 and Fig 1). Two separate interactions occurred at the Lime Point site in September and November of 2016. Of the 3 companies contacted for information, 2 shared data and 1 declined to respond. Consequently, no additional data is available regarding the incidents that occurred at Raynor Site and Lime Point.

Age was estimated for 6 whales; there were 5 subadults and one calf. Four of these age estimates were inferred by body length estimates of 910–1051 cm, consistent with subadult humpback whales. Sex was determined for two of the eight whales, and both were female.

Timing of humpback whale interactions varied interannually. In five cases, only a single interaction was reported per year (Table 1), but in 2016, three interactions occurred in a 3-month span (September–November). Cumulatively, four interactions occurred in November, two occurred in March, one in September, and one in December (Table 1). Five of the eight interactions occurred between December 1 and January 31 (winter), more than in any other season. Humpback whale sightings also varied interannually. During peak season (April–October) [2] humpback whales are sighted all over BC waters, but in off season (November–March) [2] humpback whales are sighted in areas closer to shore and up inlets (Fig 2).

Not all sites were active at the time the interaction occurred (Table 2). The Mussel Rock, Lime Point, and Wehlis Bay facilities were fallow when the humpback whale entered the site; whereas, the Raynor, Gold River, Ross Pass, and Millar Channel incidents were in production. Activity at each site with respect to smolt size, the use of underwater lights and bubble curtains is presented in Table 2.

**Table 2:** Aquaculture operations at the time of humpback whale interactions.

Date	Location	Operations	Smolt size stocked	Lights	Bubble curtain	Time of discovery	Damage done	Notes
Nov. 1, 2008	Raynor Site	Active	300 g smolts	—	—	—	—	—
Mar. 25, 2009	Mussel Rock	Fallow	None	None	None	10:35 am	—	A predator net had been deployed the previous day in two pieces and was to be sewn together by divers the day that the whale was observed
Mar. 27, 2013	Ross Pass	Active	Smolts in smolt nets (1” mesh)	Yes, underwater	None	5:50 am	—	No whale observed during an inspection at 11 pm the night before
Sep. 12, 2016	Lime Point	Fallow	—	—	—	—	—	—
Nov. 15, 2016	Lime Point	Fallow	—	—	—	—	—	—
Nov. 25, 2016	Gold River	Active	Smolts, size not indicated	Unknown	None	—	—	—
Dec. 2, 2018	Millar Channel	Fallow	Smolts in smolt nets (1” mesh)	Yes, underwater (dusk–dawn)	Yes, in use at time of incident	8:00 am	Hole at 23 m depth	—
Nov. 14, 2021	Wehlis Bay	Active	None	None	None	8:00 am	1.5 m x 1.5 m hole at 28 m depth	Site was in the process of being deactivated. The containment nets had been removed and only the predator net was installed and fully weighted

The depth at incident sites ranged from 10–140 m. Defects were observed in the predator nets of two out of eight facilities at depths of 23–35 m.

### Case summaries

**Fig 3:**
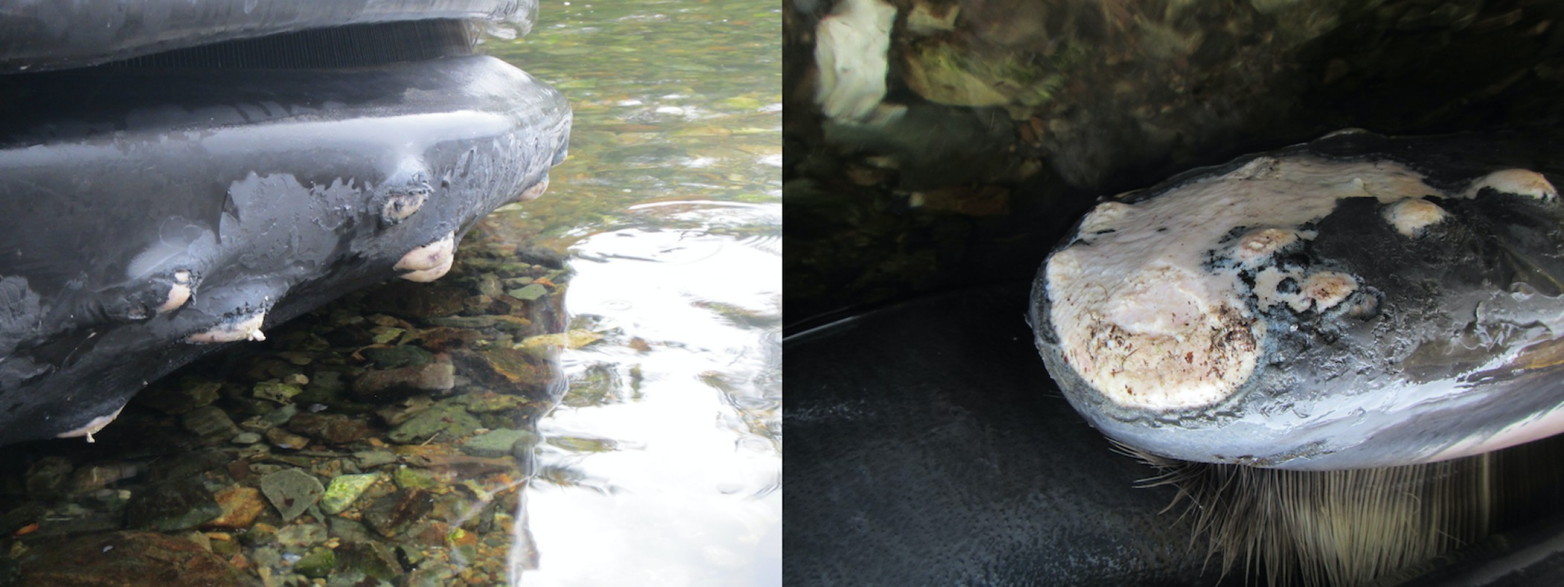
Tubercle and rostrum abrasions on a deceased humpback whale found at an aquaculture facility in Gold River, BC. The images are printed under a CC BY license, with permission from Paul Cottrell, original copyright 2023.

**Fig 4:**
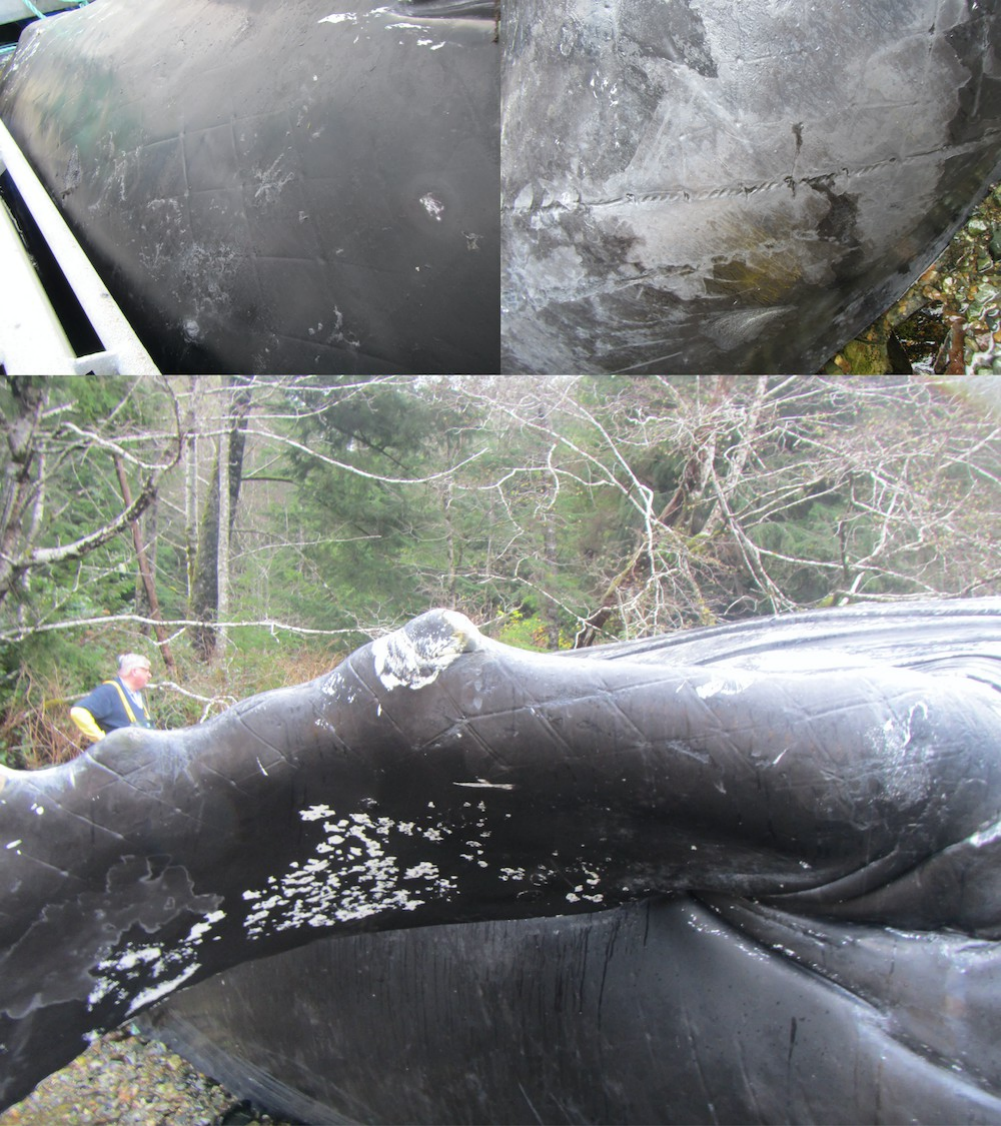
Chain impressions on the skin of a deceased humpback whale found at an aquaculture facility in Gold River, BC. Top two images show impressions on the body and lower image shows impressions on the leading edge of the left flipper. The images are printed under a CC BY license, with permission from Paul Cottrell, original copyright 2023.

1. On Nov. 1, 2008, a subadult humpback whale was found inside a containment net at Raynor Site. Divers were called in immediately and the whale was coaxed out of the pen unharmed. The whale caused a 2 x 2.5 m defect in the bottom panel of the net pen. The hole was covered with a seine net. Divers report that many fish were in the pen, but an unknown number had escaped.
2. On Mar. 25, 2009, a humpback whale calf was reported inside the predator net at Mussel Rock. Net pens had not yet been installed. With the whale swimming in the enclosure, one end of the net was dropped, and the whale swam out. An adult female humpback whale was seen outside the net and both animals swam away together.
3. On Mar. 25, 2009, a subadult female humpback whale in good body condition was found dead and floating between the containment and predator net of a salmon farm in Ross Pass, near Ahousaht. A necropsy and enforcement investigation were unable to determine if the whale died on site or if it died and subsequently entered the site via prevailing currents. Post-mortem change hampered microscopic review of the sampled tissues and precluded precise determination of a cause of death. There are three proposed theories: (1) The whale was dead and submerged prior to the net breach and passively forced it through the net at depth by currents, (2) The whale was dead prior to the net breach and subsequent putrefaction increased its buoyancy and penetrated through the net by upward momentum, (3) The whale was alive when it penetrated the net, and subsequently died.
4. On Sep. 12, 2016, a humpback whale of unknown age became entangled in an anchor support line at an inactive aquaculture facility at Lime Point. There were multiple wraps through the baleen. BCMMRN responded and were able to disentangle and release the whale.
5. On Nov. 15, 2016, a subadult humpback whale was reported entangled in an anchor support line and died at a fallow aquaculture facility at Lime Point near Klemtu, Bella Bella. The features of this whale were distinct to the animal released on Sep. 12, 2016. Samples were taken from the whale but were not analyzed. No diagnostic pathology was performed.
6. On Nov. 25, 2016, a subadult female humpback whale in moderate body condition was reported dead at an aquaculture facility in Gold River. The whale was initially observed alive between the containment and predator nets and did not appear to be entangled. In an attempt to release the whale, the predator net was dropped ~30 m and the animal was subsequently not observed, until the carcass refloated, and the predator net was raised. Evidence from the necropsy was consistent with the whale becoming entangled and subsequently drowned in the dropped netting. Skin lesions were consistent with net impressions as well as rope and chain entanglements (Figs 3 and 4). Associated hemorrhage indicated that the injuries had occurred antemortem.
7. On Dec. 2, 2018, a humpback whale of unknown age was found between the predator and containment nets at the Millar Channel aquaculture facility. The predator net was cut, and the whale successfully released.
8. On Nov. 14, 2021, a subadult humpback whale was found swimming between the predator and containment nets at the Wehlis Bay aquaculture facility. The predator net was dropped to a depth of ~25 m and then the whale was subsequently seen swimming out of the predator net. On Nov. 16, 2021, divers inspected the predator net and identified a 2 m x 2 m defect at a depth of 30 m.

## Discussion

### Results summary

From 2008–2021, eight humpback whale interactions with BC salmon farms were reported to the BCMMRN, consisting of three lethal entanglements and five nonlethal disentanglements. Without human intervention, it is likely that the entangled animals would have died. Based on reviewing the available information, the majority of these incidents: (1) involved subadult whales, (2) occurred in November, (3) were related to predator nets, and (4) had successful outcomes with human intervention.

### Pathology associated with entrapment

In one incident, the humpback whale was entrapped in the net and the cause of death was attributed to drowning. Evidence to support this includes skin and blubber lesions with associated hemorrhage consistent with net, rope, and chain entanglement (Figs 3 and 4). Similar lesions have been documented in aquaculture net entangled humpback whale calf [22]. Although the cutaneous lesions would likely not have resulted in the death of the animal, sequelae from exertion, metabolic acidosis, musculoskeletal damage, and hypoxia may have contributed to antemortem morbidity. Documenting these lesions is valuable for future identification of aquaculture entanglements that may have been lethal or where an animal was successfully released or escaped.

### Marine mammal interactions with aquaculture

Documented interactions between marine mammals and aquaculture are a common occurrence, but tend to involve pinnipeds, and odontocetes rather than mysticetes [23]. Large aggregates of fish confined in net pens may entice predators which likely view them as a reliable and accessible food source. Scavenger fish also aggregate around fish farms because of nutrients in the water from leftover feed, feces, and organic matter removed from the nets, and the shelter provided by the equipment [24, 25]. The abundance of both farmed and wild fish in a localized area appears to attract marine mammals, especially otariids (sea lions and fur seals) [17, 18, 26, 27], but also odontocetes [28], benthic invertebrates and birds [29].

Pinnipeds account for 1–12% of salmon losses at net-pen facilities worldwide, primarily due to predation [26]. Consequently, fish farms employ measures to prevent predation of stock, such as predator nets, acoustic deterrents, and lethal removal [30]. Over the study period (2008–2021), 839 other marine mammal fatalities occurred at BC aquaculture facilities with a disproportionate number of California sea lions (*n* = 472) and harbour seals (*n* = 358) [23]. These numbers likely represent the relative abundance of these species along the BC coast and propensity to acclimate to human activities. In comparison, humpback whale fatalities at aquaculture sites (*n* = 3) are rare and not previously documented within the region. As pinniped-aquaculture interactions are more common than reported whale interactions with farms, few guidelines that directly address large whales have been developed [31]. Despite the lack of guidelines for preventing and dealing with humpback whale interactions, five humpback whales entangled within anchor lines, predator nets, or net pens were successfully released. With any future aquaculture incidents involving large cetaceans, consultation with the BCMMRN or individuals experienced with disentanglements is recommended.

In other countries, associations between common bottlenose dolphins and aquaculture facilities are reported. For example, direct predation on production fish has been recorded in Hawaii where at least one dolphin has performed actions that result in fish escapement from cages, on more than one occasion [28]. These interactions have been documented since 2008 and include about one quarter of the Hawaii Island Resident dolphin population, suggesting that associating with fish farms is a successful foraging strategy. In contrast, humpback whales in BC do not appear to reside for extended periods [2] or form long-term associations with aquaculture facilities (as evidenced by the complete lack of reported observations).

Humpback whale-aquaculture interactions are not unique to BC. Humpback whales have also been documented as entangled or entrapped in aquaculture equipment in Chile [22, 32], Iceland [33], and Australia [34]. Both events in Chile occurred at salmon farms and described humpback whales entangled in predator nets. One event involved a calf that died [22]. The second incident was an adult whale that was entrapped in the predator net of an inactive facility [32]. The event took place in December and workers at the salmon farm were able to release the whale by dropping the predator net. Although there are too few data to infer a seasonality, anecdotally, the timing (December) and circumstances (inactive facility) are similar to documented cases in BC.

### Contributing factors

This study identified five factors associated with two or more of the incidents reported: facility design, environmental features, seasonality, humpback whale age, and humpback whale feeding behaviour.

#### Aquaculture facility design

Most salmon farms in BC have similar designs consisting of cage arrays with multiple containment pens for salmon, surrounded by an external net to exclude or restrict access of salmon predators, such as seals, sea lions, sharks, river otters and mink. These floating arrays are anchored but move up and down with the tides. In this report, entanglement in the predator nets of salmon farms was observed in five out of eight cases. From the event descriptions, humpback whales appear to have perforated the base of the net to enter the enclosure. Once inside the predator net, whales can access the surface to breathe but are unable to find an exit. Predator nets are designed to deter smaller marine mammals, such as seals and sea lions, but do not have the tensile strength to exclude large whales. Large cetaceans and in particular, humpback whale interactions with aquaculture facilities are rare, and have not been recognized or accounted for in facility design in BC.

In this case series, two humpback whales were entangled in anchor support lines at a fallow aquaculture facility. Like fishing gear (crab and fishing lines), support lines can get entangled in the mouths of whales or wrapped around the tail stock and eventually entrap animals. The degree of movement and access to air depends on the anatomic location of the gear, how tightly it is wrapped, and whether or not it is anchored [35, 36]. A whale that gets caught in a line will attempt to swim away, which can further constrict the line and cause the whale to become even more entangled [37, 38].

Both anchor support line entanglements in this series occurred at the same aquaculture facility, which was in the process of being decommissioned. After the first incident, all anchor support lines were scheduled for removal, but a second whale entanglement occurred before the lines were to be removed. Subsequent to the second incident, a change in policy was issued through DFO that all anchor support lines at fallow sites were to be removed from BC waters to prevent further entanglements (Cottrell [Unpublished]). Since their removal in 2016, there have been no further humpback whale-anchor support line entanglements reported.

#### Environmental features

Most of the fish farms involved in humpback whale interactions were in channels, with shallow depths and net pens situated near shore. In late fall, herring, a frequent prey of humpback whales, transit through narrow channels to get to shallow inshore waters [39]. Humpback whales spend time in channels with one hypothesis that they enter and range up channel to follow fish there. Humpback whales may target herring in channels during the winter months, causing them to approach salmon farms.

Site depths ranged from 10–140 m and provided some space for humpback whales to swim beneath the fish farms even with predator nets installed. Due to a lack of sonar and anatomic positioning of the eyes, baleen whales may not detect a net above them causing them to accidentally strike and penetrate the bottom of the predator nets. We suspect that the whales are approaching fish farms in search of herring. Fish farms may be an abundant food resource because they also attract small schooling fish [24, 25]. This may be particularly enticing in the winter months when the distribution of humpback whale prey likely changes, and access to previously reliable resources diminishes. As a result of prey shifts, humpback whales may forage in different locations seasonally (Fig 2), which may bring them closer to aquaculture facilities increasing the likelihood of net-pen encounters.

Aquaculture facilities are located in nearshore sheltered areas that humpback whales are known to frequent (Fig 1) [1]. However, the highest densities of humpback whales in BC are found in deeper waters, offshore (Fig 1) [7–10, 40], suggesting that the realized overlap in habitat use is minimal. It is important to note however that most studies of the distribution and abundance of humpback whales in BC do not survey the inlets and nearshore waters where aquaculture facilities are located. Moreover, there are insufficient farm observations and inconsistent surveillance efforts for statistical analyses or modelling. Thus, these results likely underestimate the true overlap in habitat use of humpback whales and fish farms. Better estimates of humpback whale presence near fish farms are needed to determine the percentage of interactions that result in entrapment and entanglement.

#### Seasonality

Despite the relatively small number of cases presented in this series, time of year is an important factor associated with humpback whale-aquaculture interactions in BC and other geographic regions. Humpback whales are present in BC year-round with a peak abundance from April to November in tandem with their annual migration from Japan, Hawaii, and Mexico to Alaska [1, 40]. Migrating humpback whales are known to feed as they transit throughout BC. Some individuals (especially juveniles) are known to reside in BC year round rather than travel to birthing regions [1]. Most incidents (5/8) occurred outside of peak humpback whale season [2], when humpback whales are sighted in areas closer to shore and up inlets (Fig 2). Sightings data also show that humpback whale habitat use shifts from deeper offshore waters in the spring and summer, to shallower nearshore waters in the winter (Fig 2). This shift may result in humpback whales spending more time near aquaculture facilities and increase the chance of an interaction occurring.

Seasonal change in habitat use may result from seasonal changes in prey distribution. Prey availability differs by season suggesting that whales may use different foraging grounds and strategies for each season. Herring is found inland in channels in the late fall and early winter [39], which is when 5 net-pen interactions occurred. These interactions coincide with seasonal changes in prey availability and suggest that time of year may be a predictor for when entanglements may occur.

Fall and winter also have the highest prevalence of storms. Storms can move or damage gear which could increase the risk of large whale entanglements, but there was no evidence that any of the sites had been damaged prior to the interactions. There was also no evidence to suggest that adverse weather contributed to the interactions.

In addition to climactic and prey considerations, all the incidents occurred in the fall, winter and spring, when the lowest numbers of humpbacks are observed in the area, and the longest nights occur. Anecdotal evidence suggests that overnight lights on fish farms attract fish and squid [29], and may have direct or indirect effects on humpback whales.

#### Humpback whale age and feeding behaviour

The total length of all whales in this case series were consistent with them being subadults. Like many young animals, subadult humpback whales may be more exploratory and less avoidant than adults [e.g., 41, 42]. This attitude may contribute to their propensity to approach salmon farms. Despite being younger, subadult humpback whales can break through ropes of similar strengths as adult humpback whales [43]. The lack of reports of adult humpback whale interactions with aquaculture in this series suggests that there may also be something unique about subadult whales.

In general, humpback whales are known for their intelligent feeding tactics and their ability to develop new tactics (e.g., trap-feeding) [44]. For example, a group of humpback whales in Alaska has been found to prey on hatchery salmon right as they are released by waiting in shallow waters nearby [45]. The initial observations of trap-feeding involved two juvenile whales, indicating that young whales may be actively involved in developing new foraging tactics, such as feeding on fish and krill aggregations near fish farm sites.

One hypothesis is that subadult whales are attracted to fish farms because of aggregation of the stocked salmon. Interestingly, in half of the incidents in this series involved fallow fish farms. Therefore, in at least half of the cases, farmed salmon did not appear to directly attract the whales to the facility. This observation highlights the importance of trying to better define environmental features or cues that may be attractants for humpback whales.

At one site, a bubble curtain was in use at the time of the incident. Humpback whales use bubbles as part of a cooperative feeding strategy to aggregate prey, termed bubble-net feeding [37]. It is uncertain if humpback whales are attracted to bubble curtains, but it is worth considering due to their increased application in aquaculture and among other ocean-based industries.

### Humpback whale interactions with other gear types

Interactions with aquaculture are estimated to account for ~6% of all humpback whale entanglements in BC (BCMMRN unpublished data). The majority of reported interactions and entanglements are with crab and prawn gear, followed by non-aquaculture netting (BCMMRN unpublished data). What is unique about aquaculture interactions is that they are consistently and accurately reported due to the comprehensive regulation of fish farms in BC. As such, the total number of incidents reported over this period is likely to be accurate. However, 6% may be an overestimate as there are likely humpback whale interactions with other gear types that go unnoticed or unreported. This can occur when a humpback whale interacts with a rope or net and frees themselves before being observed. Humpback whale interactions with aquaculture may be less prevalent than entanglement in crab and prawn gear because unlike active aquaculture facilities which are tightly regulated and monitored, crab and prawn gear is usually left unattended and sometimes derelict. In addition, crab and prawn gear tends to be more abundant, and is usually put out in the summer months when humpback whales are more abundant.

### Entanglement and entrapment response

The humpback whale-aquaculture interactions reported here highlight the need for aquaculture workers to diligently observe, report and consult with the BCMMRN prior to releasing humpback whales entrapped in predator nets. Humpback whale entanglements are complex incidents that require expert intervention. In five cases, aquaculture workers were able to drop the predator net low enough for the whale to swim out of the facility. Although a simple procedure, there is the potential for adverse outcomes as shown for one incident in this series when the whale become entangled and drowned in a lowered predator net. This specific incident was avoidable and emphasizes the need to consult with experts in the event of a humpback whale gaining access to a net-pen facility.

### Recommendations to prevent humpback whale-aquaculture interactions and improve outcomes for entangled whales

Several lessons can be learned from the lethal and nonlethal humpback whale-aquaculture interactions described herein. Humpback whale interactions are currently impossible to predict but can be managed successfully by informed responders. Thus, we recommend that aquaculture and stranding response coordinators develop protocols to prepare for large whale accessing net-pen facilities and the potential for entanglements. Protocols should detail responses to large whales present in the vicinity of a farm, in terms of monitoring and who to inform and seek support when an entanglement has occurred. Similar recommendations have been made by Eynon [46].

In BC, late fall and winter were identified as the most common seasons for humpback whale interactions with aquaculture to occur. Increased awareness monitoring, possibly by communicating with the BC Cetacean Sighting Network, or passive acoustic monitoring could alert workers and marine mammal managers of potential threats and interactions.

## Conclusion

Humpback whales have interacted with aquaculture facilities in BC as early as 2008, and possibly before. These interactions have resulted in injury and death to a small number of whales and caused damage to aquaculture gear that allowed fish to escape. The documented entrapment and entanglement of young humpback whales in predator nets of Atlantic salmon farms shows the whales are usually still able to breathe, are good candidates to mount a response, and should have a good prognosis for release unharmed. In this case series, there are too few animals to infer a specific cause of what attracted these young whales to fish farms. We nevertheless suspect they were foraging for prey given that many of the reported interactions occurred outside peak humpback whale abundance when there may be a shortage of prey or simply something better elsewhere. Humpback whale interactions with aquaculture are not entirely preventable, but on-site and public education as well as implementing a response plan may improve the number of successful live outcomes.
